# CDH1 Gene and Hereditary Diffuse Gastric Cancer Syndrome: Molecular and Histological Alterations and Implications for Diagnosis And Treatment

**DOI:** 10.3389/fphar.2018.01421

**Published:** 2018-12-05

**Authors:** Wenyi Luo, Faysal Fedda, Patrick Lynch, Dongfeng Tan

**Affiliations:** ^1^Department of Pathology, The University of Texas MD Anderson Cancer Center, Houston, TX, United States; ^2^Department of Gastroenterology, Hepatology and Nutrition, The University of Texas MD Anderson Cancer Center, Houston, TX, United States

**Keywords:** CDH1 cadherin-1 gene, lobular breast cancer, management, familial gastric cancer (FGC), signet ring cell carcinoma

## Abstract

Gastric cancer, a group of common malignancies, results in the most cancer mortality worldwide after only lung and colorectal cancer. Although familial gastric cancers have long been recognized, it was not until recently that they were discovered to be associated with mutations of specific genes. Mutations of *CDH1*, the gene encoding E-cadherin, are the most common germline mutations detected in gastric cancer and underlie hereditary diffuse gastric cancer (HDGC) syndrome. All reported HDGCs are the pure diffuse type by Lauren classification and are associated with dismal prognosis once the tumor invades the submucosa. Because *CDH1* germline mutations are inherited in an autosomal-dominant fashion and have high penetrance, the International Gastric Cancer Linkage Consortium (IGCLC) developed criteria to facilitate the screening of *CDH1* mutation carriers; these criteria have been proven to have excellent sensitivity and specificity. Recent histologic studies suggest that HDGC progresses through several stages. Even when the tumor becomes “invasive” in lamina propria, it may stay indolent for a long time. However, the molecular mechanisms that induce the transitions between stages and determine the length of the indolent phase remain to be determined. Although the standard management for *CDH1* mutation carriers is prophylactic total gastrectomy, many questions must be answered before the surgery can be done. These include the optimal surveillance strategy, the best strategy to choose surgical candidates, and the ideal time to perform surgery. In addition to increasing the risk of gastric cancer, *CDH1* germline mutations also increase the risk of invasive lobular carcinoma of the breast, and possibly colorectal adenocarcinoma, and are associated with blepharocheilodontic syndrome (a congenital development disorder). However, the optimal management of these conditions is less established owing to insufficient data regarding the risk of cancer development. This review focuses on molecular and histological findings in HDGC, as opposed to sporadic diffuse gastric cancer, and their implications for the management of *CDH1* mutation carriers and the diagnosis and treatment of HDGC. Other conditions associated with *CDH1* germline mutations and future research directions are also discussed.

## Overview of Hereditary Diffuse Gastric Cancer

Gastric cancer is the fifth most common malignancy in the world and the third leading cause of cancer death in both sexes worldwide after lung cancer and colorectal cancer (Ferlay et al., 2013). Although the overall incidence and mortality of non-cardia gastric cancer has declined over the past four decades, the rates are increasing among persons younger than 50 years (Wang et al., 2018); these increases are associated with the rapidly increased recognition of diffuse gastric cancer from 1978 to 2000, after this recognition decreased slightly from 2001 to 2005 (Wu et al., 2009). Gastric cancer in the young is associated with a high incidence of poorly-differentiated and signet ring cell morphology and demonstrates advanced stage at presentation and poor survival even with surgical intervention (Rona et al., 2017).

Familial clustering of gastric cancer has long been noticed (Maimon and Zinninger, 1953). Approximately 8–30% of gastric cancer patients have a positive family history (van der Post et al., 2015a). However, not all these cancers are hereditary. Countries with a high incidence of sporadic gastric cancer, such as Japan and Korea, have a lower frequency of germline mutations in familial gastric cancers than low-incidence countries do (Lee et al., 2014). The cause of familial clustering in high-incidence countries is more likely environmental than hereditary.

Approximately 1–3% of gastric cancers are truly hereditary (Fitzgerald et al., 2010). The underlying genetic alteration in 60% of cases remains unknown (Gaston et al., 2014). Gastric cancer predisposition has been linked to familial cancer syndromes, including Lynch syndrome (Capelle et al., 2010), Peutz-Jeghers syndrome (van Lier et al., 2010), Li-Fraumeni syndrome (Masciari et al., 2011), familial adenomatous polyposis syndrome (Fornasarig et al., 2018) and recently described gastric adenocarcinoma and proximal polyposis syndrome of the stomach (Worthley et al., 2012). Similar to sporadic gastric cancer, gastric cancer in these syndromic patients can be either intestinal or diffuse type. Diffuse type gastric cancer does not appear to be overrepresented in these syndromes (Fewings et al., 2018). A higher frequency of deleterious germline *ATM* mutations has been detected in gastric cancer patients; however, the histologic types have not been studied (Huang et al., 2015). Gastric cancer containing a significant diffuse component can occur in patients bearing certain germline mutations. For example, *MAP3K6* germline mutations have also been associated with familial gastric cancer, and the gastric cancers associated with *MAP3K6* predominantly have a signet ring cell morphology, although a minor glandular component has been described (Gaston et al., 2014). Some syndromic patients develop pure diffuse type gastric cancer, referred to as hereditary diffuse gastric carcinoma (HDGC). In addition to *CDH1*, the gene encoding E-cadherin, germline pathogenic variants in *PALB2* and other cancer-predisposing genes have been identified by whole-exome sequencing of HDGC families (Fewings et al., 2018). The clinical implications of these genes remain to be elucidated.

HDGC accounts for 1–3% of gastric cancers (Guilford et al., 1998). Although *CDH1* somatic mutations are present in up to 50% of sporadic diffuse gastric carcinoma (SDGC) (Becker et al., 1994) and epigenetic inactivation of *CDH1* had been detected in several tumor types (Yoshiura et al., 1995), it was not until 1998 that Guilford (Guilford et al., 1998) reported the presence of a *CDH1* mutation in a large kindred from New Zealand with early-onset diffuse gastric cancer. This seminal study, for the first time, established the pathogenic role of *CDH1* mutations in HDGC. *CDH1* germline mutations are detected in approximately 25% of HDGC patients and are inherited in an autosomal-dominant fashion (Caldas et al., 1999). The estimated cumulative incidence of gastric cancer by age 80 years is 70% in male carriers and 56% in female carriers. In addition to having an increased risk of gastric cancer, *CDH1* mutation carriers also have an increased risk of lobular breast carcinoma. The estimated cumulative incidence of lobular breast cancer in female carriers is as high as 60% (Fitzgerald et al., 2010; Guilford et al., 2010; Hansford et al., 2015). HDGC patients with germline *CDH1* mutations have lower 1 and 5 years survival rates (36 and 4%, respectively) than HDGC patients without germline *CDH1* mutations do (48 and 13%, respectively) (van der Post et al., 2015a), emphasizing the importance of screening for *CDH1* germline mutations (Benusiglio et al., 2015).

The International Gastric Cancer Linkage Consortium (IGCLC) defined the clinical criteria to select patients eligible for *CDH1* germline mutations: (1) two or more documented cases of gastric cancer in first- or second-degree relatives regardless of age, with at least one confirmed diffuse gastric cancer; (2) diffuse gastric cancer before age 40 years without a family history; or (3) families with diagnoses of both diffuse gastric cancer and lobular breast carcinoma, at least one before age 50 years (van der Post et al., 2015b). These criteria have a sensitivity of 0.79–0.89, specificity of 0.70, positive predictive value of 0.14–0.19, and negative predicative value of 0.97 (Benusiglio et al., 2015; van der Post et al., 2015a). One study showed that HDGC patients without a known *CDH1* mutation diagnosed by multigene cancer panel before surgery were more likely to have metastatic disease and die of their disease than were patients with known *CDH1* mutation status (Moslim et al., 2018), suggesting that genetic counseling and detection of *CDH1* mutations in asymptomatic carriers improves HDGC patient survival.

This review will discuss current molecular and histological findings in HDGC, as opposed to SDGC, and their implications for the management of *CDH1* mutation carriers and the diagnosis and treatment of HDGC. Other conditions associated with *CDH1* germline mutations and future research directions will also be discussed briefly.

## Molecular Pathogenesis, Cellular Origin, and Initiation

The gene *CDH1*, the coding gene for E-cadherin, is located on chromosome 16q22.1 and consists of 16 exons (Berx et al., 1995). More than 100 *CDH1* pathogenic germline variants have been described in HDGC families (Hansford et al., 2015), and they are scattered across the entire gene, including introns and each of 16 exons (Corso et al., 2012; Melo et al., 2017; Li et al., 2018). A minority (27%) of the reported pathogenic mutations have been reported in multiple families, likely owing to a common ancestor or mutation in hot spots (Hansford et al., 2015; Li et al., 2018). Cases of sporadic gastric cancer with pathologic germline mutations have also been reported (Garziera et al., 2013). Overall, the most common mutations are small insertions and deletions (35%). Other mutations include nonsense mutations (16%), splice site mutations (16%), and large exon deletions and missense mutations (28%) (Guilford et al., 2010). Phenotype is not correlated with the location or type of germline *CDH1* mutation (Guilford et al., 2010). In particular, genotype is not correlated with the presence of lobular breast cancer in HDGC families (Schrader et al., 2008).

E-cadherin is a member of the cadherin family, which consists of a group of glycoproteins that mediate cell-cell adhesion in a calcium-dependent manner (Takeichi, 1991). Mature E-cadherin has an ectodomain consisting of five tandem repeats, a transmembrane domain, a single transmembrane domain, and a cytoplasmic domain (Takeichi, 1995). The extracellular domain is critical for cell-cell adhesion, correct folding, and dimerization (Shapiro et al., 1995; Nagar et al., 1996). The cytoplasmic domain of E-cadherin interacts with β-, p120-, and α-catenins anchored to the actin cytoskeleton (Blaschuk et al., 1990). The interaction with actin is required for membrane deformation processes such as endocytosis, exocytosis, autophagy and receptor/channel recycling, and is involved in cell membrane maintenance, tension, ion channel activity among others (Gumbiner, 1996; Godwin et al., 2018). E-cadherin deficiency undermines the efficiency of these different processes and potentially cell survivability (Godwin et al., 2018). E-cadherin plays an important role in blastomere adhesion during development, which polarizes the cells and allows differentiation to occur (Fleming et al., 1992). In normal adult tissue, E-cadherin is involved in the maintenance and homeostasis of the epithelium (Gumbiner, 1996). In addition to its structural role, E-cadherin can also transduce signals from the extracellular domain through the cytoplasmic tail into the nucleus to alter gene expression (Bershadsky, 2004). The reduction or complete absence of E-cadherin, which has been detected in many cancer types, is associated with loss of epithelial morphology and increased invasiveness through epithelial-mesenchymal transition (Berx et al., 1998; Machado et al., 1999) and is correlated with high grade, advanced stage, and poor prognosis (Guilford, 1999).

Reduced or absent E-cadherin expression is seen in both the *in situ* and invasive components of HDGC, suggesting that inactivation of E-cadherin is an early event (Carneiro et al., 2004). E-cadherin loss generally correlates with the identification of an alteration of the second *CDH1* allele (i.e., a second hit) (Barber et al., 2008a). The most frequent second hit inactivation mechanism is *CDH1* promoter hypermethylation, which occurs in approximately 50% of primary tumors, whereas a second mutation or loss of heterozygosity is less frequently identified (Grady et al., 2000; Oliveira et al., 2009). The methylation is allele-specific and is uncommon in HDGC patients without *CDH1* germline mutations (Barber et al., 2008a). In patients with *CDH1* germline mutations, methylation occurs only when the wild-type allele is methylated (Grady et al., 2000). The trigger of the second hit remains unclear. The normal expression of E-cadherin in tissue between tumor foci and the variable expression levels of E-cadherin between HDGC tumor foci suggest that these tumor foci are multiclonal and develop independently (Charlton et al., 2004). Therefore, certain environmental factors affecting the entire gastric mucosa may be present as a trigger. However, the well-characterized risk factor for sporadic gastric cancer, *Helicobacter pylori*, together with other lesions commonly seen in the background of sporadic gastric cancer, are rarely detected in the total gastrectomy specimens from asymptomatic HDGC patients (Carneiro et al., 2004; Humar and Guilford, 2009; Rocha et al., 2018).

Different from other tumor repressors, complete loss of *CDH1* expression is not sufficient for the development of invasive carcinoma, as has been demonstrated in transgenic animal models. Conditional knockout of *CDH1* in mouse stomach induces signet ring–like cells in stroma (analogous to intramucosal signet ring cell carcinoma) but not the development of carcinoma invading into submucosa (Mimata et al., 2011). Other modifying genes, such as *Smad4* and *p53*, are required for aggressive diffuse gastric cancer or metastasis to occur in mice (Pereira et al., 2006; Park et al., 2014, 2018). Similar findings were also reported in lobular mammary carcinoma (Derksen et al., 2006). These findings recapitulated those in humans, in which lesions of various morphologies are seen, with some confined within basement membrane (meeting the conventional definition of carcinoma *in situ*), some existing in the lamina propria, and others invading into submucosa. Intramucosal carcinoma can remain indolent for a long time before submucosal invasion and lymph node metastasis ensue (van der Post et al., 2016). Therefore, the progression of HDGC is most likely a multi-stage process, with the initial loss of E-cadherin enabling tumor cells to detach from basement membrane and the subsequent loss of other modifying genes rendering the cells truly invasive. In humans, different or additional molecular mechanisms might be involved. C-Src kinase, a well-characterized inducer of epithelium mesenchymal transition, was found to be differentially expressed and activated in signet ring cells at different stages: C-Src was strongly expressed in poorly-differentiated and dedifferentiated cells in the mucosal layer and in the cells invading the muscularis mucosae but was not expressed in intramucosal signet ring cells. Consistent with this, downstream targets of c-Src, such as fibronectin, Fak, and Stat3, were differentially activated (Humar et al., 2007).

The upregulation of c-Src in HDGC has been linked to the loss of inhibition of epidermal growth factor receptor (EGFR), the upstream tyrosine receptor kinase of c-Src. E-cadherin has an inhibitory effect on EGFR, and the effect relies on the integrity of the extracellular domains of E-cadherin (Qian et al., 2004). Cell lines derived from HDGC patients with impaired extracellular domains of E-cadherin were less able to suppress EGFR signaling than cell lines with wild type E-cadherin were (Mateus et al., 2007). Loss of EGFR inhibition increases the activation of EGFR and its downstream components, such as phosphoinositide 3-kinase and c-Src. This theory is supported by the finding that some HDGC-derived cell lines demonstrate sensitivity to EGFR inhibition (Li et al., 2018).

Interestingly, studies also support a functional interaction between HER2 and the E-cadherin through interactions with β-, p120-, and α-catenins which leads to a decrease of the E-cadherin-mediated cell adhesion and facilitates tumor cell invasion and migration. Association between specific CDH1 polymorphisms with a subset of HER2-positive gastric cancer and possibly favorable prognosis has been described (Caggiari et al., 2017).

There are multiple theories regarding the cell of origin of signet ring cell carcinoma in HDGC patients. Gastric stem cells are candidates because they reside in the upper neck region (Karam et al., 2003), where HDGC seems to originate (Humar et al., 2007). An epithelial origin has also been suggested, and direct conversion from gastric epithelium to mucous containing signet ring cells is believed to be the first step of carcinogenesis (Charlton et al., 2004). A neuroendocrine cell of origin has also been proposed because normal neuroendocrine cells of the upper gastrointestinal tract lack E-cadherin expression (Waldum et al., 2014). This proposal explains the discrepancy between the lack of atypia and malignant biological behavior. Studies in a *CDH1* knock-out animal model showed that parietal cells can “float” in the lamina propria, mimicking signet ring cell carcinoma, suggesting that parietal cells are possible cell of origin for signet ring cell carcinoma (Mimata et al., 2011).

## Histopathology and Progression Model

No gross lesion can be detected in the early stages of disease (Rogers et al., 2008). Advanced HDGC demonstrates linitis plastica (Guilford et al., 2010). Owing to the lack of a gross lesion, histologic examination of the entire grossly normal gastric mucosa of the prophylactic gastrectomy specimen is still the standard practice for asymptomatic *CDH1* mutation carriers (Corso et al., 2014). Careful examination can identify signet ring cell carcinoma (mostly multifocal and intramucosal) as well as signet ring carcinoma *in situ* in over 90% of these specimens (Corso et al., 2014).

Between 0 and 200 cancer foci have been detected in prophylactic gastrectomy specimens. The sizes of the foci vary from 0 to 14 mm. No correlations between the number or location of foci identified and the age or sex of the patients or their specific germline mutations have been identified (Barber et al., 2008b). The topological mapping of tumor foci in gastrectomy specimens to assess the feasibility of targeted biopsies has yielded mixed results. Two studies showed that most cancer foci were concentrated in the proximal stomach (Rogers et al., 2008; Black et al., 2014). Similarly, in another study of seven patients, the majority of foci were identified in the fundus (44.7% of all foci) and body (40.2%), and all patients had lesions in these two areas (Barber et al., 2008b). Another study based on 6 fully mapped cases revealed predominant localization of tumor foci in the distal stomach body-antral transitional zone (Charlton et al., 2004). No topographic association was found in a different study (Huntsman et al., 2001).

Because of the considerable time commitment, researchers have sought methods to facilitate the detection of foci of signet ring cell carcinoma. Periodic acid-Schiff (PAS) staining is superior to hematoxylin and eosin (HE) staining for screening prophylactic gastrectomy specimens from *CDH1* mutation carriers (Lee et al., 2010). In contrast to HE staining, PAS staining increases the contrast between signet ring cells (which show magenta cytoplasm in PAS staining) and lamina propria (PAS-negative) and therefore significantly reduces the screening time and number of missed foci.

All reported gastric cancers identified in HDGC families are the pure diffuse type by Lauren classification. No pathogenic germline mutations have been found in families with the intestinal, medullary, or mixed types (van der Post et al., 2015a). Although signet ring cell morphology is common, especially if the tumor is intramucosal, poorly-differentiated carcinomas without signet ring cell features are also seen (Guilford et al., 2007; Humar et al., 2007). Various histological morphologies have been observed in lesions containing signet ring cells, including signet ring cell carcinoma *in situ*, in which the signet ring cells are confined within the epithelium by basement membrane; pagetoid spread, in which the signet ring cells spread below the preserved epithelium of glands/foveolae without breaking the basement membrane (essentially another form of signet ring cell carcinoma *in situ*); and invasive carcinoma (Huntsman et al., 2001; Carneiro et al., 2004).

Because it frequently occurs distant from the intramucosal carcinoma, signet ring cell carcinoma is probably a distinctive lesion instead of colonization of the epithelium by invasive carcinoma (Carneiro et al., 2004). Many intramucosal signet ring cell carcinoma foci in preventive gastrectomy specimens do not have an adjacent *in situ* component. The discrepancy between the numerous invasive carcinoma foci and the low number of *in situ* carcinoma lesions suggests that signet ring cell carcinoma *in situ* is not an obligated precursor of invasive carcinoma in HDGC (Milne et al., 2007). The process of basement membrane breakthrough, which is still hypothetical, may be induced by the expression of type IV collagenases, which have been found to be upregulated when E-cadherin is downregulated through the inactivation of the other copy of *CDH1* gene (Margulis et al., 2005). Although the secretion of these enzymes is limited, it is sufficient for cells to penetrate the basement membrane in the absence of adhesion and polarity (Humar and Guilford, 2009).

Several morphologies are seen in tumor cells outside the basement membrane. Two morphological populations of signet ring cells are present in the intramucosal carcinoma. “Well-differentiated large cells” are signet ring cells with abundant mucin and eccentrically located flattened nuclei with mild atypia. They are positive for mucicarmine and pCEA and mostly located beneath the surface epithelium. “Small cells” are signet ring cells with less mucin and have hyperchromatic and atypical nuclei. They are located in the neck region and are rarely positive for mucicarmine or pCEA (Lee et al., 2018). The small cells have the highest proliferative index, which is similar to that of normal gastric cells in the upper neck region. Immunofluorescence studies have also demonstrated that the base of the intramucosal carcinoma in HDGC has a proliferative index and a differentiation marker expression profile similar to those of the upper neck region of normal gastric units which is therefore the likely origin of the disease (Humar et al., 2007). The locations of large and small cells and their differences in proliferation suggest an initial upper migration stage when maturation occurs.

The small cells can be further classified as well-differentiated or poorly-differentiated, with the latter showing nuclear and cytoplasmic reactivity to p16 immunohistochemical staining and displaying more aggressive behavior. Most of the foci studied have demonstrated combined morphology (Lee et al., 2018). Despite the seemingly different differentiation, both large and small cells express the epithelial markers cytokeratin 8 and 18 but not markers of epithelial-mesenchymal transition such as vimentin, high Ki67 (Barber et al., 2008b), and c-Src (Humar et al., 2007). Conversely, epithelial-mesenchymal transition, the hallmark feature once the tumor invades the submucosa, is associated with poor differentiation and increased proliferation. This is achieved by increased activation of c-Src kinase and its downstream targets fibronectin, Fak, and Stat3 (Humar et al., 2007). The long and asymptomatic presence of intramucosal carcinoma in *CDH1* mutation carriers and the low proliferative index suggest that intramucosal carcinoma has an indolent nature. However, the trigger of the progression from well-differentiated cells to poorly-differentiated cells and further to submucosal invasion is still unknown. The small cells, particularly the poorly-differentiated small cells, are morphologically similar to metastatic lobular carcinoma of the breast. When breast carcinoma is a diagnostic possibility, immunohistochemical staining for estrogen receptor, progesterone receptor, GATA3 and gross cystic disease fluid protein-15, among others, may be used to aid in the differential diagnosis. The mentioned immunohistochemical stains are most commonly positive in metastatic lobular carcinoma of the breast (Kim et al., 2018), however a panel approach is preferred.

Several patterns of E-cadherin have been detected immunohistochemically, including complete loss of staining, attenuated staining, and aberrant staining. However, screening by E-cadherin immunohistochemical staining is not feasible, because 60% of gastric cancers without E-cadherin expression and 70% of gastric cancers with aberrant expression are negative for *CDH1* alterations (Corso et al., 2013).

Although morphologically similar, HDGC and SDGC are different histologically and immunohistochemically. Signet ring cell carcinoma *in situ*, including pagetoid spread of signet ring cells, appears specific to HDGC with *CDH1* mutations, as it has not been reported in SDGC (Carneiro et al., 2004; Fitzgerald et al., 2010) or HDGC without germline *CDH1* mutations (van der Post et al., 2015b). The presence of signet ring carcinoma *in situ* cells should trigger genetic testing for possible HDGC. The tumor cells in HDGC patients are negative for CDX2, whereas most SDGC cases (with one exception), have shown positive CDX2 expression (Lee et al., 2018), implying that HDGC and SDGC have different pathogeneses. Accordingly, the absence of CDX2 in signet ring cell carcinomas in patients without a family history may prompt genetic screening for HDGC.

Background alterations in the gastric mucosa of HDGC include infrequent *Helicobacter pylori* and intestinal metaplasia. Other changes include mild chronic gastritis, foveolar hyperplasia, tufting, vacuolization of superficial epithelium, and fundic and hyperplastic polyps (Carneiro et al., 2004; Rocha et al., 2018). Mimickers of signet ring cell carcinoma, including clear changes, globoid changes, xanthomatous cells, and pseudo–signet ring cells associated with lymphoid aggregates, are also seen (Rocha et al., 2018).

## Other Disease Association

Female kindreds of an HDGC family have increased risk of invasive lobular carcinoma, with a lifetime risk of 50–60% (Fitzgerald et al., 2010; Guilford et al., 2010; Hansford et al., 2015). Invasive lobular carcinoma may be the first manifestation of HDGC (Benusiglio et al., 2013). Invasive lobular carcinoma was also recently discovered in *CDH1* germline mutation carriers who never developed HDGC (Corso et al., 2016). The hallmark molecular alterations of all lobular neoplasia (atypical lobular hyperplasia, lobular carcinoma *in situ*, and invasive lobular carcinoma) are loss of cellular adhesion and loss or decreased expression of E-cadherin (Zou et al., 2009). Although atypical lobular hyperplasia and lobular carcinoma *in situ* are considered markers of increased risk of sporadic invasive lobular carcinoma, their roles and characteristics in invasive lobular carcinoma in *CDH1* germline mutation carriers have rarely been studied. One recent study showed that up to 8% of patients with bilateral LCIS have germline mutations in *CDH1* (Petridis et al., 2014). Earlier studies showed a lower prevalence of *CDH1* germline mutations in patients with no history of gastric carcinoma (Rahman et al., 2000; Masciari et al., 2007).

HDGC syndrome is also possibly associated with colorectal carcinoma because colorectal carcinoma has been observed in HDGC families, and loss of E-cadherin has been detected in both the tumor and adjacent normal colonic tissue. In one study, a *CDH1* missense germline mutation co-segregated with colorectal carcinoma; however, the same mutation was also present in the normal population. Interestingly, the colorectal adenocarcinoma associated with HDGC syndrome is not necessarily signet ring cell carcinoma; instead, it can be intestinal adenocarcinoma (Salahshor et al., 2001). One case of an appendiceal signet ring cell carcinoma occurring with a gastric intramucosal signet ring cell carcinoma has been reported (Hamilton et al., 2013).

*CDH1* mutations have also been identified in patients with blepharocheilodontic syndrome, a congenital development disorder causing dysmorphic features, which can be accompanied by imperforate anus, hypothyroidism, and neural tube defect. However, the mutations identified in blepharocheilodontic syndrome all occur in extracellular calcium binding repeats and are functionally distinct from those identified in HDGC (Kievit et al., 2018).

## Clinical Management

Guidelines recommend genetic screening offered from the age of consent (16–18 years) (van der Post et al., 2015b). Factors such as the emotional and physical health of the individual and the earliest age of gastric cancer in the family should be considered (Guilford et al., 2010). With newer technologies, screening of HDGC carriers is becoming more efficient. A next-generation sequencing panel covering all 16 exons has been developed and validated (El-Husny et al., 2016). Liquid biopsies based on cell-free circulating DNA have been used to detect *CDH1* promoter methylation in the plasma/serum of gastric cancer patients (Tsujiura et al., 2014).

*CDH1* mutation types are important in determining the management of patients at risk. Truncating germline mutations are deleterious, and a total prophylactic gastrectomy should be offered. However, the management of carriers of missense mutations is not straightforward, and the burden is to prove or disprove pathogenic relevance. In contrast to SDGCs, which have mutations clustered in exons 7 and 9, HDGC has no hotspot for germline mutations. Only 17% of germline mutations are shared by more than one family or isolated individual (Suriano et al., 2006). Therefore, the pathogenic relevance of most missense mutations has to be individually validated. This can be achieved using computational methods including frequency in normal controls, co-segregation, recurrence, and *in silico* tools such as structural modeling and SIFT software in combination with databases containing *CDH1* sequencing data, such as The Exome Variant Server of the University of Washington and the variant database http://www.LOVD.nl/CDH1 (Suriano et al., 2006; van der Post et al., 2015a). For difficult cases, functional assessment, such as *in vitro* evaluation of an E-cadherin–induced cell adhesion and invasion assay, may be ultimately needed to ascertain the role of *CDH1* missense mutations (Suriano et al., 2006; Barber et al., 2008b; Corso et al., 2011). A functional cell model (Suriano et al., 2003) and several animal models (Pereira et al., 2006; Caldeira et al., 2009) have been established for this purpose.

The recommended management for HDGC patients is prophylactic total gastrectomy (van der Post et al., 2015b). Prophylactic gastrectomy carries a 3–6% mortality rate and a 100% morbidity rate owing to eating habit changes, dumping syndrome, diarrhea, and weight loss (Lewis et al., 2001). Although guidelines recommend prophylactic gastrectomy for germline *CDH1* mutation carriers in their 20 or 30 s, the optimal time for gastrectomy is debatable and should be individualized. Carriers can develop advanced gastric carcinoma as early as age 14 years or may never develop cancer owing to incomplete penetrance, which occurs in 20–30% of *CDH1* germline mutation carriers. The onset age among families varies widely; this variation may be caused by different types of mutations, with missense mutations having less penetrance. Even within the same family, cancer develops at different ages, probably owing to when and how the second allele is inactivated. It has been recommended that prophylactic gastrectomy may be considered at an age younger than that of the youngest affected person in the family but should not be considered for family members in whom a causative mutation has not been identified or who have less penetrant forms of susceptibility to gastric cancer (Huntsman et al., 2001).

Endoscopic surveillance is needed if gastrectomy is contraindicated owing to comorbidity, if the patient is younger than the age recommended for surgery, or if the patient refuses surgery. The probability of detecting intramucosal carcinoma depends on the total number of lesions for a given total area of abnormal mucosa. The probability is higher for a large number of small lesions. The probability of detecting intramucosal carcinomas by five blind random biopsies has been estimated to be lower than 5% in cases with small numbers of lesions (Carneiro et al., 2004). In one theoretical estimation, 1,768 biopsies are needed to assure a 90% rate of detecting at least 1 cancer focus (Fujita et al., 2012). Current guidelines recommended that individuals be offered annual high-definition white light endoscopy (van der Post et al., 2015b). Chromoendoscopy with Congo red/methylene blue, which had been used successfully to detect lesions harboring tumor foci, has been discontinued owing to concerns about Congo red toxicity (Shaw et al., 2005). In addition to sampling endoscopically visible lesions, random sampling covering the pre-pyloric area, antrum, transitional zone, body, fundus, and cardia is also recommended (van der Post et al., 2015b). A minimum of 30 biopsies is recommended, as described in the Cambridge protocol (Fitzgerald et al., 2010). It is possible to increase the yield of signet ring cell carcinoma by adhering strictly to the Cambridge protocol. Using careful white-light examination with targeted biopsies and 24 random biopsies (4 each of the prepylorus, antrum, T zone, body, fundus, and cardia) combined with detailed histopathology, Lim et al (van der Post et al., 2015a) found signet ring cell carcinomas in 14 of 22 patients with, and 2 of 7 patients without, *CDH1* mutations fulfilling the 2010 HDGC criteria.

Surgical intervention for HDGC is no different from that for sporadic gastric cancer. Current guidelines recommend a total gastrectomy with Roux-en-Y reconstruction (van der Post et al., 2015a). A D1 lymph node dissection can be considered because most tumors in HDGC patients are at least T1a, and the presence of T1b lesions cannot be ruled out preoperatively (van der Post et al., 2015a). The management of gastric heterotopia has been controversial. Theoretically, signet ring cell carcinoma can arise from any gastric mucosa, including ectopic gastric mucosa and this forms the basis of the recommendation that all gastric mucosa should be removed during surgery (van der Post et al., 2015b). In some institutions, including MD Anderson Cancer Center, absence of gastric mucosa (including ectopic gastric mucosa) at the margins is required and can be confirmed by frozen section (van der Post et al., 2015a).

Because of the high lifetime risk of invasive lobular carcinoma, women with *CDH1* mutations should undergo annual radiologic surveillance, with bilateral breast magnetic resonance imaging being the preferred modality, along with annual clinical breast examination (van der Post et al., 2015b). The suggested age at which breast surveillance should start varies from 25 to 35 years, whereas the role of chemoprevention remains unclear (Cisco and Norton, 2008; van der Post et al., 2015b; Corso et al., 2016; Wright et al., 2018). On the other hand, prophylactic bilateral mastectomy is not routinely recommended, and breast surveillance is preferred (Corso et al., 2014). Contralateral mastectomy for *CDH1* mutation carriers diagnosed with invasive lobular carcinoma may be considered on an individual basis (Wright et al., 2018).

## Author Contributions

DT conceived, designed, and reviewed the paper; WL and FF wrote the paper; PL reviewed clinical management.

### Conflict of Interest Statement

The authors declare that the research was conducted in the absence of any commercial or financial relationships that could be construed as a potential conflict of interest.
